# Supplementary material for: Risk and symptoms of COVID-19 in health professionals according to baseline immune status and booster vaccination during the Delta and Omicron waves in Switzerland—A multicentre cohort study
Source: PLoS Med. 2022 Nov 7;19(11):e1004125. doi: 10.1371/journal.pmed.1004125 (PMC9678290; doi:10.1371/journal.pmed.1004125)
Supplement: S1 STROBE Checklist — (PDF) [file pmed.1004125.s001.pdf]

STROBE Statement—Checklist of items that should be included in reports of ***cohort studies***

	Item No	Recommendation	Page No
**Title and abstract**	1	(*a*) Indicate the study's design with a commonly used term in the title or the abstract<br>(*b*) Provide in the abstract an informative and balanced summary of what was done and what was found	See title (a) and abstract (b).
**Introduction**			
Background/rationale	2	Explain the scientific background and rationale for the investigation being reported	Background, paragraphs 1 and 2
Objectives	3	State specific objectives, including any prespecified hypotheses	Background paragraph 3
**Methods**			
Study design	4	Present key elements of study design early in the paper	Methods, section "Study design and population"
Setting	5	Describe the setting, locations, and relevant dates, including periods of recruitment, exposure, follow-up, and data collection	Methods, section "Study design and population", Figure 1
Participants	6	(*a*) Give the eligibility criteria, and the sources and methods of selection of participants. Describe methods of follow-up<br>(*b*) For matched studies, give matching criteria and number of exposed and unexposed	a) Methods, section "Study design and population"<br>b) not applicable
Variables	7	Clearly define all outcomes, exposures, predictors, potential confounders, and effect modifiers. Give diagnostic criteria, if applicable	Methods, section "SARS-CoV-diagnostics"; "Definition of predictor variables"; "Outcomes", also Table S1 and Figure S1
Data sources/ measurement	8*	For each variable of interest, give sources of data and details of methods of assessment (measurement). Describe comparability of assessment methods if there is more than one group	Table S1
Bias	9	Describe any efforts to address potential sources of bias	Validation of self-reported swabs: Methods, section "SARS-CoV-diagnostics";<br>Confounding: Methods, section "Statistical analysis regarding infection risk"<br>Definition of viral periods (sensitivity analysis): Methods, section "Statistical analysis regarding infection risk"
Study size	10	Explain how the study size was arrived at	Not applicable (NA)
Quantitative variables	11	Explain how quantitative variables were handled in the analyses. If applicable, describe which groupings were chosen and why	Table S1

Statistical methods	12	(*a*) Describe all statistical methods, including those used to control for confounding<br>(*b*) Describe any methods used to examine subgroups and interactions<br>(*c*) Explain how missing data were addressed<br>(*d*) If applicable, explain how loss to follow-up was addressed<br>(*e*) Describe any sensitivity analyses	a) Methods, section "Statistical analysis regarding infection risk"; "Supplementary analyses"; "Frequency of SARS-CoV-2 symptoms"; also Supplements<br>b) Methods, section "Supplementary analyses"<br>c) Methods, section "Statistical analysis regarding infection risk", paragraph 2; also Supplements<br>d) not done<br>e) Methods, section "Statistical analysis regarding infection risk", paragraph 2 (viral period); Sensitivity analyses regarding imputation of missing values are described in Supplements
**Results**			
Participants	13*	(a) Report numbers of individuals at each stage of study—eg numbers potentially eligible, examined for eligibility, confirmed eligible, included in the study, completing follow-up, and analysed<br>(b) Give reasons for non-participation at each stage<br>(c) Consider use of a flow diagram	a) Results, section "Study population"; also Figure S2<br>b) not done<br>c) Figure S2
Descriptive data	14*	(a) Give characteristics of study participants (eg demographic, clinical, social) and information on exposures and potential confounders<br>(b) Indicate number of participants with missing data for each variable of interest<br>(c) Summarise follow-up time (eg, average and total amount)	a) Results, section "Study population"; also Table 1<br>b) Table 1<br>c) Table 1
Outcome data	15*	Report numbers of outcome events or summary measures over time	Results, section "SARS-CoV-2 (re)-infections by immune status and time period"

Main results	16	(*a*) Give unadjusted estimates and, if applicable, confounder-adjusted estimates and their precision (eg, 95% confidence interval). Make clear which confounders were adjusted for and why they were included<br>(*b*) Report category boundaries when continuous variables were categorized<br>(*c*) If relevant, consider translating estimates of relative risk into absolute risk for a meaningful time period	a) For risk of infection, unadjusted: Table S2 and Figure 2;<br>adjusted: Table 2 and Figure 2<br>For symptoms, unadjusted: Results, section "SARS-CoV-2 symptoms according to immune status and time period"; adjusted: same section, plus Table S7 and Table S8<br>b) NA<br>c) not done
Other analyses	17	Report other analyses done—eg analyses of subgroups and interactions, and sensitivity analyses	Results, section "Multivariable analysis and sensitivity analyses" and section "Supplementary analyses", see also Tables S3 to S6.
**Discussion**			
Key results	18	Summarise key results with reference to study objectives	See Discussion, first paragraph
Limitations	19	Discuss limitations of the study, taking into account sources of potential bias or imprecision. Discuss both direction and magnitude of any potential bias	See Discussion, paragraph 8
Interpretation	20	Give a cautious overall interpretation of results considering objectives, limitations, multiplicity of analyses, results from similar studies, and other relevant evidence	See Discussion, paragraph 2 (hybrid immunity), paragraph 3 (vaccination only), paragraph 4 (infection only), paragraph 5 (vaccine type), paragraph 6 (booster vaccination), paragraph 7 (symptoms)
Generalisability	21	Discuss the generalisability (external validity) of the study results	See Discussion, paragraph 8 (limitations)
**Other information**			
Funding	22	Give the source of funding and the role of the funders for the present study and, if applicable, for the original study on which the present article is based	See submission system

*Give information separately for exposed and unexposed groups.

Note: An Explanation and Elaboration article discusses each checklist item and gives methodological background and published examples of transparent reporting. The STROBE checklist is best used in conjunction with this article (freely available on the Web sites of PLoS Medicine at http://www.plosmedicine.org/, Annals of Internal Medicine at http://www.annals.org/, and Epidemiology at http://www.epidem.com/). Information on the STROBE Initiative is available at http://www.strobe-statement.org.
