# Supplementary material for: Risk and symptoms of COVID-19 in health professionals according to baseline immune status and booster vaccination during the Delta and Omicron waves in Switzerland—A multicentre cohort study
Source: PLoS Med. 2022 Nov 7;19(11):e1004125. doi: 10.1371/journal.pmed.1004125 (PMC9678290; doi:10.1371/journal.pmed.1004125)
Supplement: S1 Methods — (PDF) [file pmed.1004125.s002.pdf]

METHODS

Missing value imputation for covariates in multivariable models

To avoid the exclusion of participants with missing values for one or several covariates, missing value imputation was performed through multivariate imputation by chained equations (R package mice, version: 3.14.0) using predictive mean matching and 10 iterations over the individual items. All time-independent covariates involved in the main models, i.e. age, sex, BMI > 30, any comorbidity, patient contact, respirator mask use, were included in the procedure. Ten series of imputations were performed, generating ten sets of covariate values, each of which was then combined with the outcomes and time-dependent covariates. Multivariable models were fitted separately to the ten data sets to obtain ten sets of model coefficients and standard errors. These were combined using the pooling rules implemented in the function "pool" of R package "mice". All results presented in tables of multivariable models are derived from the pooled statistics.

Verification of proportional-hazard assumption in Cox regression

The proportional-hazard assumption was checked using scaled Schoenfeld residuals from the main multivariable model (Table 2). For each variable in the model, plots of Schoenfeld residuals against time were examined for patterns, and score tests for deviations from the proportional-hazard assumption (i.e. for time-dependence of the regression coefficients) were performed with the function cox.zph in R package survival, version: 3.1-12). All variables fulfilled the assumption in the model for the Delta period. In the model for the Omicron period, the assumption was violated for the effect of booster vaccination. After subdivision of this term into booster effects on events observed before and after 15 February (which was close to the median date of the events observed during the Omicron period), the proportional-hazard assumption was fulfilled.
