# Supplementary material for: Risk and symptoms of COVID-19 in health professionals according to baseline immune status and booster vaccination during the Delta and Omicron waves in Switzerland—A multicentre cohort study
Source: PLoS Med. 2022 Nov 7;19(11):e1004125. doi: 10.1371/journal.pmed.1004125 (PMC9678290; doi:10.1371/journal.pmed.1004125)
Supplement: S1 Table — (PDF) [file pmed.1004125.s003.pdf]

Table S1. Co-variable definitions, levels, and time points when variables were obtained.

Variable name (unit)	Definition	Levels	Time points
Anthropometrics/baseline health			
Age (years)	Age at time of last serology	Number	Sept 21
Body mass index (kg/m2)	Body mass index at time of study entry	Number	Sept 20
Gender		Female; Male (Ref[a])	Sept 20
Comorbidities	Presence of any of the following: arterial hypertension, diabetes, cancer, pulmonary disease, rheumatologic disease, other	Yes; No (Ref)	Sept 20
SARS-CoV-2 exposures and risk behaviors			
Patient contact	Being involved in patient care	Yes; No (Ref)	Sept 21
Mask type	Mask type preferentially used during COVID-19 patient contact during study period (outside of aerosol-generating procedures)	Always respirator; mixed/mostly surgical masks (Ref)	Sept 21
Positive household contact[b]	At least one household contact with positive SARS-CoV-2 swab in current month (as reported by participants)	Yes; No (Ref)	Sep 21 – Mar 22
Any negative test[b]	Having had at least one negative nasopharyngeal swab (PCR or rapid antigen test) in last month	Yes; No (Ref)	Sep 21 – Mar 22
SARS-CoV-2 vaccinations and infections			
Baseline vaccinations	Type and date of any SARS-CoV-2 vaccines received before September 2021		Sep 20 - Sep 21
Booster or follow-up vaccinations[b]	Type and date of any SARS-CoV-2 vaccines received after September 2021		Sep 21 – Mar 22
Time since immunization[b]	Calculated in months since immunization event		calculated
SARS-CoV-2 infections (predictor variable)	Date of any SARS-CoV-2 infections before September 2021		Sep 20 - Sep 21
SARS-CoV-2 infections (main outcome)	Date and symptoms[c] of any SARS-CoV-2 infections after September 2021		Sep 21 – Mar 22

[a] Ref, Reference
[b] Time-dependent variables in models
[c] Symptoms include coryza, headache, cough, muscle/limb pain, loss of smell or taste, chills, body temperature over 38° Celsius (self-measurement), nausea, dizziness, chest pain, diarrhea
