# Supplementary material for: Risk and symptoms of COVID-19 in health professionals according to baseline immune status and booster vaccination during the Delta and Omicron waves in Switzerland—A multicentre cohort study
Source: PLoS Med. 2022 Nov 7;19(11):e1004125. doi: 10.1371/journal.pmed.1004125 (PMC9678290; doi:10.1371/journal.pmed.1004125)
Supplement: S2 Table — (PDF) [file pmed.1004125.s004.pdf]

Table S2. Hazard ratios with 95% confidence intervals from separate univariable Cox regression models for each predictor involved in the multivariable models regarding COVID-19 risk by period.

	Delta		Omicron	
	HR (95% CI)	p	HR (95% CI)	p
Group V (vs. N)	0.33 (0.22-0.50)	<0.001	0.57 (0.40-0.79)	0.001
Group I (vs. N)	0.25 (0.11-0.57)	0.001	0.75 (0.47-1.22)	0.249
Group H (vs. N)	0.04 (0.02-0.10)	<0.001	0.35 (0.24-0.51)	<0.001
Age (per decade)	0.93 (0.81-1.06)	0.290	0.76 (0.71-0.82)	<0.001
Male vs. female	1.10 (0.76-1.58)	0.624	0.87 (0.70-1.07)	0.195
Body mass index > 30 kg/m2	0.75 (0.45-1.26)	0.282	0.94 (0.73-1.21)	0.631
Patient contact	0.73 (0.52-1.03)	0.075	0.94 (0.77-1.15)	0.542
Respirator mask use	0.77 (0.51-1.16)	0.206	0.95 (0.77-1.17)	0.607
Positive household	10.61 (7.87-14.29)	<0.001	6.48 (5.51-7.61)	<0.001
Any negative test in last month	1.52 (1.12-2.08)	0.008	1.25 (1.05-1.48)	0.010
Booster	0.36 (0.18-0.72)	0.004	0.68 (0.58-0.81)	<0.001

N (no immunity): No reported infection and anti-N/-S negative and no previous SARS-CoV-2 vaccination; V (vaccinated): no reported infection and anti-N negative, but twice vaccinated; I (infected): infection reported or anti-N positive (at any time), but no vaccination; H (hybrid immunity): reported infection or anti-N positive (at any time) and vaccination (≥1 dose).

HR, Hazard Ratio; CI, Confidence Interval

For results of multivariable analysis, please refer to Table 2 in the main text.
