# Supplementary material for: Risk and symptoms of COVID-19 in health professionals according to baseline immune status and booster vaccination during the Delta and Omicron waves in Switzerland—A multicentre cohort study
Source: PLoS Med. 2022 Nov 7;19(11):e1004125. doi: 10.1371/journal.pmed.1004125 (PMC9678290; doi:10.1371/journal.pmed.1004125)
Supplement: S3 Table — (PDF) [file pmed.1004125.s005.pdf]

**Table S3.** Sensitivity analyses: Comparison of adjusted hazard ratios obtained in Cox models with missing value imputation (pooled results from ten imputations, as presented in Table 2 of main document), without missing value imputation (i.e. complete case analysis), and with exclusion of events occurring during the period of variant overlap between 6 December 2021 and 3 January 2022.

| **Delta period** | Original result (Table 2) | No missing value imputation | Without variant overlap |
|---|---|---|---|
| Group V (vs. N) | 0.47 | 0.45 | 0.45 |
| Group I (vs. N) | 0.26 | 0.22 | 0.24 |
| Group H (vs. N) | 0.06 | 0.06 | 0.06 |
| Age (per decade) | 0.97 | 0.93 | 1.01 |
| Male vs. female | 1.16 | 1.14 | 1.27 |
| Body mass index > 30 kg/m2 | 0.84 | 0.89 | 0.82 |
| Patient contact | 0.77 | 0.79 | 0.73 |
| Respirator mask use | 0.99 | 0.98 | 1.04 |
| Positive household | 9.66 | 9.92 | 8.92 |
| Negative test in last month | 1.26 | 1.19 | 1.18 |
| Booster | 0.42 | 0.43 | 0.38 |
| **Omicron period** | | | |
| Group V (vs. N) | 0.85 | 0.84 | 0.90 |
| Group I (vs. N) | 0.74 | 0.73 | 0.83 |
| Group H (vs. N) | 0.52 | 0.50 | 0.57 |
| Age (per decade) | 0.79 | 0.79 | 0.79 |
| Male vs. female | 0.87 | 0.88 | 0.85 |
| Body mass index > 30 kg/m2 | 1.02 | 1.02 | 1.04 |
| Patient contact | 0.89 | 0.87 | 0.89 |
| Respirator mask use | 1.09 | 1.11 | 1.08 |
| Positive household | 6.17 | 6.16 | 6.20 |
| Negative test in last month | 1.11 | 1.13 | 1.10 |
| Booster | 0.81 | 0.82 | 0.83 |

N (no immunity): No reported infection and anti-N/-S negative and no previous SARS-CoV-2 vaccination; V (vaccinated): no reported infection and anti-N negative, but twice vaccinated; I (infected): infection reported or anti-N positive (at any time), but no vaccination; H (hybrid immunity): reported infection or anti-N positive (at any time) and vaccination (≥1 dose).
