# Supplementary material for: Risk and symptoms of COVID-19 in health professionals according to baseline immune status and booster vaccination during the Delta and Omicron waves in Switzerland—A multicentre cohort study
Source: PLoS Med. 2022 Nov 7;19(11):e1004125. doi: 10.1371/journal.pmed.1004125 (PMC9678290; doi:10.1371/journal.pmed.1004125)
Supplement: S4 Table — (PDF) [file pmed.1004125.s006.pdf]

Table S4. Model with time-dependent effect of booster in the Omicron-dominant period.

	aHR and 95% CI	p-value
Group V (vs. N)	0.84 (0.58–1.22)	0.364
Group I (vs. N)	0.70 (0.43–1.14)	0.149
Group H (vs. N)	0.51 (0.35–0.76)	0.001
Age (per decade)	0.79 (0.73–0.85)	<0.001
Male vs. female	0.86 (0.69–1.06)	0.158
Body mass index > 30 kg/m2	1.03 (0.80–1.32)	0.846
Patient contact	0.89 (0.72–1.10)	0.279
Respirator mask use	1.12 (0.90–1.40)	0.300
Positive household	5.63 (4.78–6.64)	<0.001
Any negative test in last month	1.09 (0.92–1.29)	0.333
Booster (events before February 15th 2022)[a]	0.60 (0.47–0.76)	<0.001
Booster (events after February 15th 2022)[b]	1.02 (0.75–1.38)	0.903

[a] Early Omicron period: Mean time from booster 35 days

[b] Late Omicron period: Mean time from booster 70 days

N (no immunity): No reported infection and anti-N/-S negative and no previous SARS-CoV-2 vaccination; V (vaccinated): no reported infection and anti-N negative, but twice vaccinated; I (infected): infection reported or anti-N positive (at any time), but no vaccination; H (hybrid immunity): reported infection or anti-N positive (at any time) and vaccination (≥1 dose). BMI, Body Mass Index.

aHR, adjusted Hazard Ratio; CI, Confidence Interval
