# Supplementary material for: Risk and symptoms of COVID-19 in health professionals according to baseline immune status and booster vaccination during the Delta and Omicron waves in Switzerland—A multicentre cohort study
Source: PLoS Med. 2022 Nov 7;19(11):e1004125. doi: 10.1371/journal.pmed.1004125 (PMC9678290; doi:10.1371/journal.pmed.1004125)
Supplement: S5 Table — Model additionally includes time from preimmunization to serology (i.e., months since last infection or vaccination) compared to the main analysis. (PDF) [file pmed.1004125.s007.pdf]

Table S5. Adjusted hazard ratios (HR) with 95% confidence intervals (CI) from multivariable Cox regression regarding risk of SARS-CoV-2 (re)-infection; group N (no immunity) excluded and group V (vaccinated) defined as reference group. Model additionally includes time from pre-immunization to serology (i.e. months since last infection or vaccination) compared to the main analysis.

	Delta		Omicron	
	aHR and 95% CI	p	aHR and 95% CI	p
Group I (vs. V)	0.27 (0.09–0.82)	0.023	0.84 (0.49–1.44)	0.527
Group H (vs. V)	0.15 (0.06–0.34)	<0.001	0.61 (0.49–0.77)	<0.001
Age (per decade)	0.93 (0.78–1.10)	0.408	0.78 (0.72–0.85)	<0.001
Male vs. female	1.21 (0.82–1.80)	0.341	0.86 (0.69–1.07)	0.173
Body mass index > 30 kg/m2	0.73 (0.40–1.32)	0.296	1.03 (0.80–1.34)	0.815
Patient contact	0.78 (0.51–1.18)	0.236	0.86 (0.69–1.07)	0.173
Respirator mask use	0.88 (0.54–1.42)	0.600	1.14 (0.91–1.43)	0.259
Positive household	10.06 (7.20–14.07)	<0.001	6.17 (5.20–7.32)	<0.001
Negative test	1.17 (0.80–1.71)	0.412	1.07 (0.90–1.28)	0.443
Months since immunisation	1.16 (1.06–1.28)	0.003	1.00 (0.96–1.04)	0.822
Booster	0.34 (0.16–0.69)	0.003	0.81 (0.65–1.03)	0.084
