# Supplementary material for: Risk and symptoms of COVID-19 in health professionals according to baseline immune status and booster vaccination during the Delta and Omicron waves in Switzerland—A multicentre cohort study
Source: PLoS Med. 2022 Nov 7;19(11):e1004125. doi: 10.1371/journal.pmed.1004125 (PMC9678290; doi:10.1371/journal.pmed.1004125)
Supplement: S6 Table — Model includes type of vaccine and timing of vaccinations between dose 1 and dose 2. (PDF) [file pmed.1004125.s008.pdf]

Table S6. Hazard ratios (HR) with 95% confidence intervals (CI) from multivariable Cox regression regarding risk of SARS-CoV-2 infection in the subgroup of those vaccinated but not infected (group V). Model includes type of vaccine and timing of vaccinations between dose 1 and dose 2.

	HR and 95% CI	p-value
mRNA-1273 vs. BNT162b2 vaccine	0.79 (0.62–1.01)	0.060
Age (per decade)	0.83 (0.75–0.90)	<0.001
Male vs. female	0.93 (0.74–1.17)	0.546
Body mass index > 30 kg/m2	0.92 (0.69–1.22)	0.556
Patient contact	0.89 (0.71–1.11)	0.292
Respirator mask use	1.06 (0.83–1.36)	0.642
Positive household	7.19 (6.00–8.63)	<0.001
Any negative test in last month	1.09 (0.90–1.32)	0.362
Time from dose 1 to dose 2 (per month)	0.96 (0.73–1.27)	0.788
Time from dose 2 to September 20th 2021 (per month)	0.96 (0.90–1.02)	0.175
Booster	0.73 (0.57–0.95)	0.018
