# Supplementary material for: Risk and symptoms of COVID-19 in health professionals according to baseline immune status and booster vaccination during the Delta and Omicron waves in Switzerland—A multicentre cohort study
Source: PLoS Med. 2022 Nov 7;19(11):e1004125. doi: 10.1371/journal.pmed.1004125 (PMC9678290; doi:10.1371/journal.pmed.1004125)
Supplement: S7 Table — Model includes only infections not preceded by booster or first vaccination. (PDF) [file pmed.1004125.s009.pdf]

Table S7. Rate ratio (RR) and 95% confidence intervals (CI) from multivariable Poisson regression regarding number of symptoms reported from SARS-CoV-2 infections during the Delta and Omicron period. Model includes only infections not preceded by booster or first vaccination.

	Rate ratio, i.e. relative number (RR and 95% CI)	p-value
Group V (vs. N)	0.94 (0.82–1.07)	0.368
Group I (vs. N)	0.91 (0.75–1.11)	0.372
Group H (vs. N)	0.81 (0.67–0.97)	0.026
Age (per decade)	1.00 (0.95–1.05)	0.983
Male vs. female	0.87 (0.75–1.01)	0.062
Body mass index > 30 kg/m2	1.16 (0.99–1.37)	0.065
Comorbidity at baseline	1.10 (0.98–1.22)	0.100
Respirator mask use	1.06 (0.92–1.23)	0.417
Time of (re)-infection (per month)	1.06 (0.99–1.14)	0.106
Omicron vs. Delta	0.81 (0.66–0.99)	0.043

N (No immunity): No reported infection and anti-N/-S negative and no previous SARS-CoV-2 vaccination; V (vaccinated): no reported infection and anti-N negative, but twice vaccinated; I (infected): infection reported or anti-N positive (at any time), but no vaccination; H (hybrid immunity): reported infection or anti-N positive (at any time) and vaccination (≥1 dose). BMI, Body Mass Index
