# Supplementary material for: Risk and symptoms of COVID-19 in health professionals according to baseline immune status and booster vaccination during the Delta and Omicron waves in Switzerland—A multicentre cohort study
Source: PLoS Med. 2022 Nov 7;19(11):e1004125. doi: 10.1371/journal.pmed.1004125 (PMC9678290; doi:10.1371/journal.pmed.1004125)
Supplement: S8 Table — Model includes booster vaccine and is therefore restricted to groups V and H. (PDF) [file pmed.1004125.s010.pdf]

Table S8. Rate ratio (RR) and 95% confidence intervals (CI) from multivariable Poisson regression regarding number of symptoms reported from SARS-CoV-2 infections during the Omicron period. Model includes effect of booster and is therefore restricted to groups V and H.

	Rate ratio, i.e. relative number (RR and 95% CI)	*p-value*
Group H vs. V	0.80 (0.71–0.91)	<0.001
Age (per decade)	0.96 (0.92–1.00)	0.074
Male vs. female	0.93 (0.83–1.05)	0.268
Body mass index > 30 kg/m2	1.03 (0.90–1.18)	0.696
Comorbidity at baseline	1.17 (1.06–1.28)	0.001
Respirator mask use	1.04 (0.92–1.17)	0.547
Time of (re)-infection (per month)	1.22 (1.13–1.31)	<0.001
Booster vaccination	0.79 (0.71–0.88)	<0.001

V (vaccinated): no reported infection and anti-N negative, but twice vaccinated; H (hybrid immunity): reported infection or anti-N positive (at any time) and vaccination (≥1 dose). BMI, Body Mass Index
