# Supplementary figures and images for: Risk and symptoms of COVID-19 in health professionals according to baseline immune status and booster vaccination during the Delta and Omicron waves in Switzerland—A multicentre cohort study
Source: PLoS Med. 2022 Nov 7;19(11):e1004125. doi: 10.1371/journal.pmed.1004125 (PMC9678290; doi:10.1371/journal.pmed.1004125)

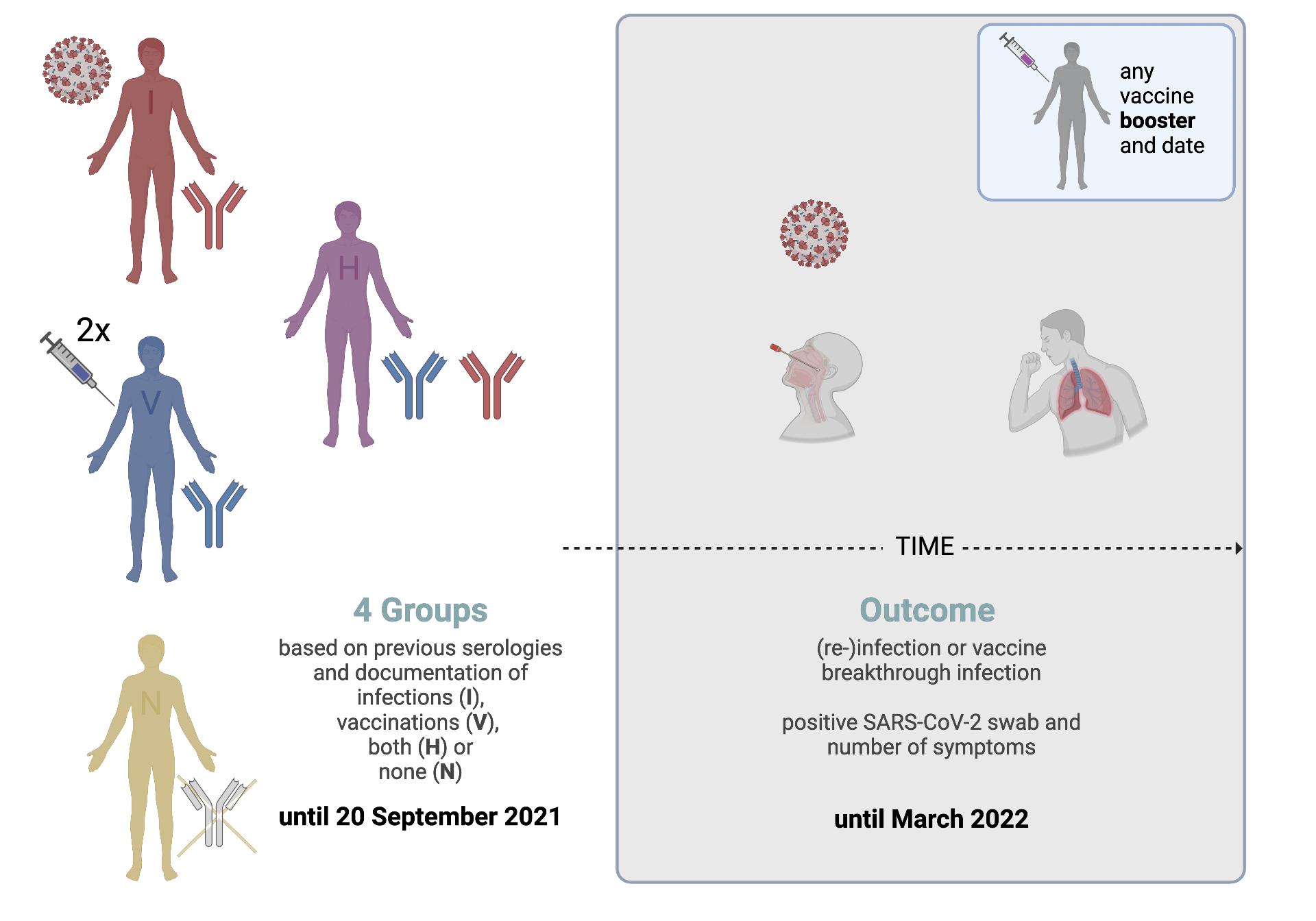

Supplement: S1 Fig — Created with BioRender.com. (TIFF) [file pmed.1004125.s011.tiff]

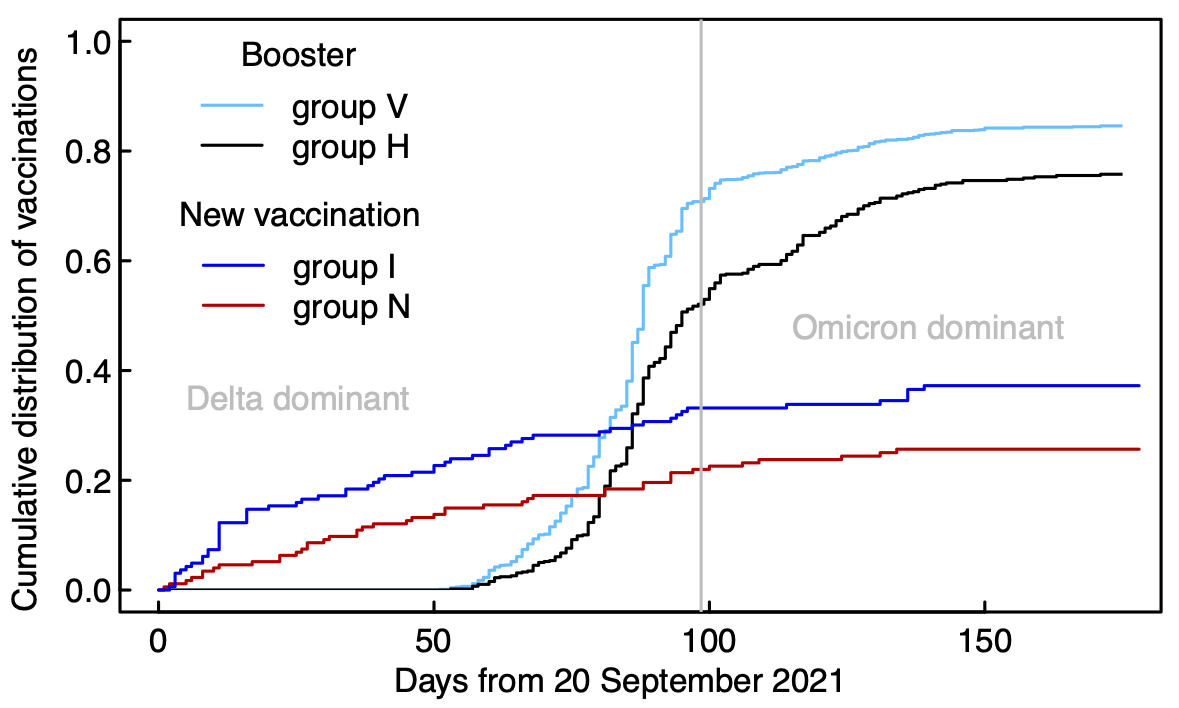

Supplement: S3 Fig — N (no immunity): no reported infection and anti-N/-S negative and no previous SARS-CoV-2 vaccination; V (vaccinated): no reported infection and anti-N negative, but twice vaccinated; I (infected): infection reported or anti-N positive (at any time), but no vaccination; H (hybrid immunity): reported infection or anti-N positive (at any time) and vaccination (≥1 dose). (TIFF) [file pmed.1004125.s013.tiff]
