# Supplementary material for: Risk and symptoms of COVID-19 in health professionals according to baseline immune status and booster vaccination during the Delta and Omicron waves in Switzerland—A multicentre cohort study
Source: PLoS Med. 2022 Nov 7;19(11):e1004125. doi: 10.1371/journal.pmed.1004125 (PMC9678290; doi:10.1371/journal.pmed.1004125)
Supplement: S2 Fig — (PDF) [file pmed.1004125.s012.pdf]

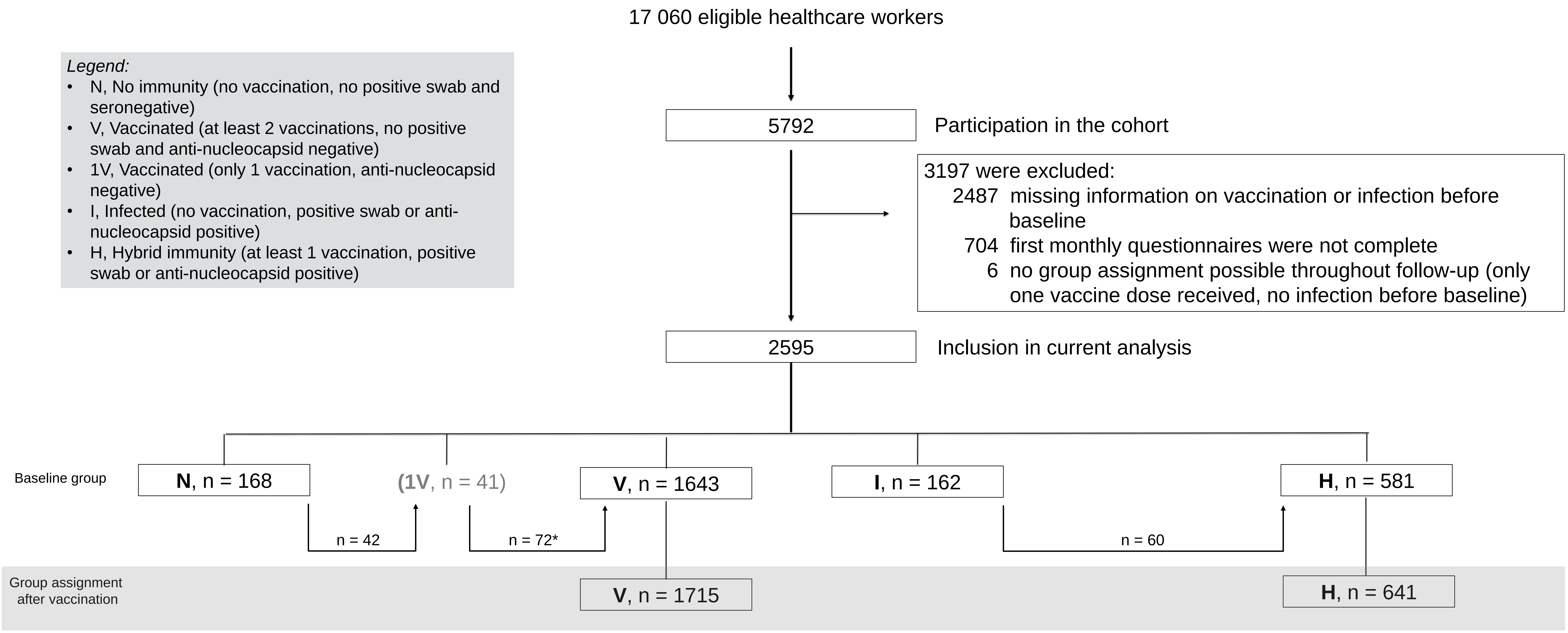

*; consisting of 31 participants from group 1V, who received an additional vaccine, and 41 participants initially from group N, who in total received two vaccinations
