# Supplementary material for: Risk and symptoms of COVID-19 in health professionals according to baseline immune status and booster vaccination during the Delta and Omicron waves in Switzerland—A multicentre cohort study
Source: PLoS Med. 2022 Nov 7;19(11):e1004125. doi: 10.1371/journal.pmed.1004125 (PMC9678290; doi:10.1371/journal.pmed.1004125)
Supplement: Attachment — Submitted filename: PMEDICINE-D-22-01834R2_RevisionNotes.pdf [file pmed.1004125.s014.pdf]

Kantonsspital St.Gallen

Kantonsspital St.Gallen

CH-9007 St.Gallen
Tel. 071 494 11 11
www.kssg.ch

Departement Innere Medizin
Infektiologie / Spitalhygiene
Prof. Dr. S. Kuster, Chefarzt

PLoS Medicine
D-22-01834R2

St.Gallen, August 30th 2022 /

Point to Point Reply for Manuscript PMEDICINE-D-22-01834R2 entitled

"Risk and symptoms of COVID-19 during the Delta and Omicron waves according to baseline immune status and booster vaccination – a prospective multicentre cohort of health professionals in Switzerland"

Dear editors
Dear reviewers

First, we would like to thank for the comments that allowed us to improve our manuscript. We have considered all comments and you will find below our point-to-point reply to the Reviews.

A major change in our manuscript is the analysis of the main outcome. As suggested by reviewer 1, we have now treated immune status as time-dependent variable. These changes came along with various adaptations in the methods, results and discussion section. The main message of the manuscript has not changed (i.e. lowest risk for those with hybrid immunity and with booster vaccination).
Also, by checking the proportional hazards assumption in one of our subanalyses, we noticed that the positive effect associated with booster vaccination was only visible in the first phase of Omicron, but not in the second. We have added this new aspect – which is very plausible when looking at the literature - to our results.

We are confident, that based on the reviewers comments and recommendations, we achieved to improve the manuscript substantially.

Christian Kahlert and Philipp Kohler, in the name of all co-authors

Request from the editors

Comments/Questions (Qe.1):

In the last sentence of the Abstract Methods and Findings section, please describe the main limitation(s) of the study's methodology."

Your study is observational and therefore causality cannot be inferred. Please remove language that implies causality, such as "Hybrid immunity and booster vaccination reduced the risk and symptom number of SARS-CoV-2 infection during Delta- and Omicron-dominant periods" (Abstract Conclusions). Refer to associations instead. Other examples include use of the term 'impact' in the final paragraph of the Introduction

Reply (Re.1):
Thank you. We agree that causal language should be removed. We have adapted the wording and highlighted the changes in the abstract and the manuscript.

In addition, we have added the following limitations to the abstract: “Important limitations are that SARS-CoV-2 swab results were self-reported and results on viral variants were inferred from the predominating strain circulating in the community at that time, rather than sequencing.

Q.e.2:
..At this stage, we ask that you include a short, non-technical Author Summary of your research…

R.e.2:
We have added an Author Summary in the manuscript after the abstract.

Q.e.3:
As noted by Reviewer #3, your Introduction does not adequately describe prior work performed in this area. Please expand your Introduction to reflect the literature more accurately

R.e.3:
We agree that other studies should be mentioned in the introduction. We have now added most of the literature suggested by reviewer 3.

Q.e.4:
Given that there are now multiple omicron subvariants in circulation, it may be useful to specify which omicron subvariant was predominant during the study period.

R.e.4:
The subvariant circulating during the “omicron period” mentioned in the manuscript was dominated by B.1.1.529.1; 21K (BA.1). This is information has been added now on line 167 (Methods, study design and population).

Q.e.5:
Consider including an additional figure to clarify the groups included in your study, as the text description can at times be difficult to follow and a figure would help the reader visualise the design.

R.e.5:
We have added an additional figure (new Figure S1) to the supplements as suggested by the editor. If the editor feels that this figure should be moved to the main manuscript, we would be happy to do so.

Q.e.6:
Please include the completed STROBE checklist as Supporting Information. Please add the following statement, or similar, to the Methods: "This study is reported as per the Strengthening the Reporting of Observational Studies in Epidemiology (STROBE) guideline (S1 Checklist).".

R.e.6:
We have now complemented the STROBE checklist guidelines and changed the sentence (lines 253ff) accordingly

Q.e.7:
When completing the checklist, please use section and paragraph numbers, rather than page numbers.

R.e.7:
This was done accordingly

Q.e.8:
Did your study have a prospective protocol or analysis plan? Please state this (either way) early in the Methods section.

R.e.8:
Indeed, there is a study protocol for the cohort of health care workers that has been approved by the ethical committee, however there was no analysis plan. This has been added to the methods section, line 164.

Q.e.9:
Table S2 ought to be moved to the main text as it is central to the interpretation of the findings.

R.e.9:
Thank you. We moved former Table S2 into the main manuscript (new Table 1)

Q.e.varia:
When reporting adjusted hazard ratios, please label these as such (aHR).

We have corrected the results accordingly.

When reporting significant findings, please quantify with both 95% CI and p-values where available.
Thank you, this has been done.

Table 1: Definitions for abbreviations do not require an accompanying flag in the table itself.
Done.

For the adjusted analyses presented in Table 1, please also present the crude analyses (unadjusted HRs) in the Supporting Information.
As requested, we added this analysis as new Table S2. Former Table 1 is now Table 2. In the caption of new Table 2 (multivariable analysis), we are now referring to new Table S2 for univariable results.

Line 245: Please update 'not anymore' to 'no longer'.
This sentence has been removed from the manuscript.

Line 251 and line 308: Please delete 'marginally' and include the p-value.
This paragraph has been rephrased.

Throughout, please ensure p-values/HRs/95% CIs are reported to the same number of decimal places between the main text and the Tables/Figures.
This has been done, thank you.

In all Tables and Figures presenting adjusted analyses, please ensure you include the variables adjusted for in the respective legends.
In Figure 2, the only adjustment made was for the effect of booster in the lower part of the table. We have clarified this directly in Figure 2. Figures 3, 4 and new Figure S3 present unadjusted results. In tables presenting multivariable Cox models, all variables involved are listed in the table and no further adjustment was made. We hope that these explanations and changes are satisfactory.

Line 280 and line 314: Please temper the use of causal language here, ('we show' could be updated to 'our observational findings suggest', or similar).
Done.

Line 289: Typo in 124,500.
Done

Line 312: Your study was not designed to assess true vaccine effectiveness, please remove this.
We have now clearly stated that vaccine effectiveness cannot be assessed with our study design. The paragraph has been adapted accordingly.

The above noted concerns regarding causal language are also applicable to your Conclusions paragraph.
We have reworded the conclusions.

Please remove details of funding, author contributions, and conflicts of interest from the Acknowledgements. This information is captured via the submission form and, in the event of acceptance, will be published as metadata.
Done.

Reviewer #1: Alex McConnachie, Statistical Review

Q1.1:

Booster vaccination is included in the models as a time-varying covariate, but individuals are censored when they are vaccinated for the first time. Could vaccination status not also be seen as a time-varying covariate in these models? Similarly, if (re)infected, could that be seen as a change of status? This would provide additional follow-up time for these individuals. I am not suggesting this as the primary analysis, but such a model could be used as a sensitivity analysis, unless there is a good reason not to do it..

R1.1:

Thank you very much for the excellent suggestions. We fully agree that it is more consistent to treat vaccination status as a time-varying covariate, as was already done for booster vaccination status. We therefore implemented the change in all analyses. This allowed us to include all individuals whose vaccination status changed before or after the start of follow-up (20 September 2021). We also updated former Table S2 (new Table 1) to reflect the status on September 20th 2021 and included the number of status changes during follow-up as additional information there. Please note that therefore the number of analyzed individuals has increased from 2451 to 2595 (for details, see text and new Figure S2). In addition, former Figures S3 and S4 were removed due to this change.

We also much appreciated the suggestion to treat (re)infections as changes of status. However, this would have implied a re-definition of our primary endpoint and a different type of analysis (multi-state or counting process instead of right-censored survival). It would also have raised new questions, e.g. whether immune status after re-infection differs from that after first infection, or whether omicron infection induces different immunity than earlier variants. Our data do not allow us to answer this question because most re-infections occurred towards the end of follow-up, and third infections were exceptional. We did therefore not implement this additional analysis in the present paper, but we shall certainly consider it in the future, with more follow-up time.

While implementing the new treatment of vaccination status, we unfortunately found an error in our previous analysis. Because booster vaccination became available in November 2021, we used 1 November as a cut-off for the identification of booster vaccination in previously vaccinated individuals (groups V and H). We erroneously applied the same criterion for the identification of new vaccinations in groups N and I. This caused us to miss new vaccinations that occurred between baseline serology and 1 November. Because this concerned multiple individuals in group I, who now changed to group H early in follow-up, the correction of this error led to some changes in our results regarding the advantage of group I over group V.

The main message of our manuscript has not changed, except that we did not observe any difference between groups I and V during the Omicron period. All results and conclusions were corrected accordingly.

Q1.2:

The adjusted regression models are based on individuals with complete baseline data only. Would multiple imputation of this missing data have been a better approach?

R1.2:

Thank you very much for the excellent suggestion, which allows us to include all individuals also in the multivariable model. We implemented it in the re-analysis of our data. We first performed three separate imputations (using the function mice in R package mice) and compared the results, as shown in the new Table S3 (sensitivity analyses). Because results of the three imputations were virtually identical (imputations mostly concerned covariates without significant effect anyway), we presented results of the first imputation as main analysis (new Table 2). For methodological details, see supplements.

Q1.3:

The analysis is repeated after exclusion of the N group. I am not sure what this achieves. Simply changing the reference group for the analysis to the V group would have done much the same thing. Looking at the coefficients in the different models, my guess is the results would be largely the same...

R1.3:
The reason is that the model without group N (former Table S3) was the basis for a sensitivity analysis that additionally included time since last immunisation (former Table S4). This variable was by nature not available for individuals of the N group.
However, we agree that for the other analyses, excluding group N does not give more information.

We have therefore decided to drop the former Table S3, but to keep former Table S4 (new Table S5) as a supplementary analysis including the effect of time since last immunization and group differences adjusted for this effect. The text in the result section has been adapted.

Q1.4:
As ever, when fitting Cox models, there is always the question of the proportional hazards assumption. How was this checked? Was the assumption acceptable?

R1.4:
The assumption was initially checked for the entire data set by testing interactions between immune status and booster vaccination and virus variants. Because these were significant, we performed and present separate analyses by virus variant. Therefore, the columns showing overall results (for Delta and Omicron period) were removed from former Table S4 (new Table S5).

The proportional hazards assumption was then checked using Schoenefeld residuals from the main multivariable model presented in new Table 2, considering residual plots and score tests for the addition of time-dependent terms implemented in the function cox.zph. Overall, the deviations from time-independence (global test) were not significant. There were weakly significant deviations in the omicron period for the effects of group and booster vaccination, which are also visible in the Kaplan-Meier plots presented in the manuscript. They are consistent with a general decrease in immunity due to vaccination or infection with time since immunisation.

We therefore added a new supplementary analysis with a time-dependent effect of booster (new Table S4). The relatively weak average effect of booster vaccination in the Omicron period (HR = 0.81 in Table 1) appeared to be caused by the transition from a stronger effect in the first part of the period (HR = 0.60) and no effect in the second part of the period (HR = 1.02).

We have added results of this supplementary analysis to the results and discussion section.

R to varia:
In order to be consistent throughout the manuscript, we have now reported HR to two decimal places. Typos have been corrected accordingly.

Reviewer #2

Q2.1:
Title: Mentions severity of COVID-19 but unclear if severity of disease is reported. Please comment on any medically attended infection, hospitalization and deaths in this cohort. Please describe symptoms in the results section or supplement.

R2.1:
Thank you for pointing this out. In fact, we have not directly looked at severity of infection, but at number of symptoms. We have therefore changed the wording in the title to "Risk and symptoms (instead of severity) of COVID-19 during the Delta and Omicron waves according to baseline immune status and booster vaccination – a prospective multicentre cohort of health professionals in Switzerland".
We unfortunately have not systematically collected data on hospitalization and death in our cohort.

Q2.2:
Line 134: If no sequencing of variants were performed, how was a positive test attributed to a specific variant?

R2.2:
As stated in the paragraph “Outcomes” of the method section, viral variants were categorized based on the community epidemiology (predominating viral strain) only and not on sequencing results. For clarification, we have added a sentence to the paragraph on SARS-CoV-2 diagnostics, referring to the paragraph Outcomes.
Because this is an important limitation, we have added a sensitivity analysis to the manuscript, where we excluded infections occurring in the period when both Delta and Omicron were circulating (December 6th 2021 to January 3rd 2022). Exclusion of the overlap period yielded similar results as the main analysis (new Table S3). This is now shown in the results and also mentioned in the discussion.

Q2.3:
Line 134: Self-reported NPS results were verified by the authors. Was this the same case for rapid antigen test at institutions that used this type of test?

R2.3:
Yes, NPS included PCRs and antigen tests. Whereas initially only PCRs were performed, antigen test became the method of choice during the pandemic. Please note that validation of NPS results was only performed at the largest participating institution. In the referred publication (Kohler et al. BMC Medicine), it reads as follows:
“Detection of SARS-CoV-2 from NPS was made by PCR or rapid antigen test, depending on the method used in the participating institutions. To verify the completeness and accuracy of self-reported NPS results, all self-reported positive tests and a random sample of negative test results were cross-checked with the database of the division of occupational health for a subgroup of HCWs from the largest participating institution.”
If reviewer 2 feels that some of this information should be included in the methods section of the current manuscript, we would agree to do so.

Q2.4:
Line 142: Please clarify "twice vaccinated". Does this mean received 2-dose primary series within recommended timeframe? received 2 doses at anytime during the study period? Etc
Twice vaccinated means two vaccine doses, irrespective of the interval between the two doses. Please note that because we have now changed our analysis and treated immune status as time-dependent variable (see answer to reviewer 1), participants were attributed to group V 7 days after receipt of the second vaccine dose. This has been clarified in the methods section.

Lines 148-149: Please clarify "single vaccine dose". It appears that Group H included at least those with 1-dose. Assuming single vaccine dose refers to vaccines that only have 1-dose but would like author's clarification.
One vaccine dose was sufficient to be classified within group H (because for previously infected individuals, one additional vaccine dose is sufficient for being considered fully vaccinated). Please note that participants almost exclusively were vaccinated with either mRNA-1273 or BNT162b2.

Q2.5:
Line 149: When was baseline serology collected? The authors mentioned periodic serology measurements earlier in the text. Please briefly include when the measurement times occurred. Due to several mentions of dates, it may be helpful to write out the full dates i.e. September 20th, 2021 instead of September 20th.
...

R2.5:
We agree that there is a need for clarification. In fact, we considered serology results from all available serology rounds performed before September 20th 2021. We have added the months of the three serology rounds in the first paragraph of the methods section.
Immune status was defined as of September 20th 2021, considering all available information from previous serologies and questionnaires. This has been clarified in the section “Definition of predictor variables” in the methods section.

Q2.6:
Lines 156-158: During variant overlap period, how were variant assignment decided for infections that occurred during circulations of both variants? I am concerned about that misclassification could impact the estimates. Please clarify how this was handled in the analysis.?

R2.6:
We agree that this could have led to misclassification, as mentioned in our limitation section. Please see our comment above. Results of the sensitivity analysis excluding infections from the overlap period showed similar hazard ratios as the main analysis.

Q2.7:
In Line 119, participants were recruited from Northern region as well. However, sequencing data was from Eastern region. Please include reasoning behind this method.

R2.7:
Thank you for this thorough review. In fact, sequencing data were collected from the North-East region. Under https://covariants.org/, Switzerland is subdivided into different regions. We took region 5 as reference, which represents all hospitals participating in our study. We have clarified this point in the methods section and changed the wording to North-Eastern in the section Outcomes of the methods.

Q2.8:
Lines 215-217: Any significant difference between groups V, I, and H?

Groups I and V were significantly different from group H but not from each other for this analysis (true for both Delta and Omicron periods).

Line 240: How were household infections data verified? Did all household contacts get tested if exposed or only if symptomatic? Please clarify in methods.

R2.8: Household infections were reported by participants. Households were not tested and reported infections were not verified. This has been specified in the results and in supplementary Table 1 (definition of predictor variables).

R to varia:
In Figure 2, the only adjustment made was for the effect of booster in the lower part of the table. We have clarified this directly in the figure.

To give the editor and the reviewers a better overview, we have summarized the new Tables and Figures in the manuscript including their relation to the original submission:

- Table 1 (former Table S2)
- Table 2 (former Table 1)
- Table S1 (former Table S1)
- Table S2 (new, results from univariable analysis)
- Table S3 (new, sensitivity analysis)
- Table S4 (new, supplementary analysis including booster as time-dependent effect)
- Table S5 (former Table S4)
- Table S6 (former Table S5)
- Table S7 (former Table S6)
- Table S8 (former Table S7)
- Former Table S3 was dropped

- Figures 1 to 4 (former Figures 1 to 4)
- Figure S1 (new)
- Figure S2 (former Figure S1)
- Figure S3 (former Figure S2)
- Former Figures S3 and S4 were dropped

Reviewer #3

Q3.1:

The authors state that "It remains currently unknown in how far natural and/or vaccine-induced immunity protect against symptomatic infection by the Omicron variant, and how this can be further mitigated by booster vaccinations" (Introduction line 101). However, in the discussion the authors go on to cite the following 5 papers, each of which report on exactly that:

1. *Effects of Previous Infection and Vaccination on Symptomatic Omicron Infections - Altarawneh et al. NEMJ 2022*
2. *Duration of mRNA vaccine protection against SARS-CoV-2 Omicron BA.1 and BA.2 subvariants in Qatar Chemaitelly et al. Nature Communications 2022*
3. *Protection with a Third Dose of mRNA Vaccine against SARS-CoV-2 Variants in Frontline Workers - Yoon et al. NEMJ 2022*
4. *Association Between 3 Doses of mRNA COVID-19 Vaccine and Symptomatic Infection Caused by the SARS-CoV-2 Omicron and Delta Variants - Accorsi et al. JAMA 2022*
5. *Covid-19 Vaccine Effectiveness against the Omicron (B.1.1.529) Variant - Andrews et al. NEMJ 2022.*

R3.1:

We acknowledge the reviewers concern. We agree that the original wording was not adequate in light of the available literature. We have therefore substantially revised the introduction, citing the above-mentioned manuscripts.

Q3.2:

There are also at least a further 4 papers that they don't cite, which add further evidence:

1. *Observed protection against SARS-CoV-2 reinfection following a primary infection: A Danish cohort study among unvaccinated using two years of nationwide PCR-test data - Michlmayr et al. The Lancet Regional Health Europe 2022*

We thank the reviewer for this reference and have added it to our manuscript, introduction (ref 12).

2. *SARS-CoV-2 Omicron Symptomatic Infections in Previously Infected or Vaccinated South African Healthcare Workers - Nunes et al. Vaccines 2022*

Thank you for pointing this out. This paper (although of small sample size) is now being cited in the Introduction, ref 22.

3. *Effectiveness of BNT162b2 Vaccine against Omicron in Children 5 to 11 Years of Age - Tan et al. NEMJ 2022*

Thank you. The paper is now cited in the Introduction, ref 21.

4. *Effectiveness of a fourth dose of covid-19 mRNA vaccine against the omicron variant among long term care residents in Ontario, Canada: test negative design study - Grewal et al. BMJ 2022*

We decided not to include this reference, because a fourth vaccination dose is not the actual topic of our analysis.

Similarly, there are many papers assessing protection against Delta, which was dominant at the beginning of the study period in this paper.

We are referring to the most important papers regarding protection against Delta in the Introduction and also in the Discussion.

Q3.3:

Overall, there's a substantial body of existing evidence on the protection against SARS-CoV-2 Delta and Omicron variants associated with prior infection and vaccination. The authors need to acknowledge this and clearly situate their study within the context of the existing literature. As a relatively small occupational cohort, it isn't clear to me that this study makes a substantial contribution to the literature, so it doesn't seem like a good fit for PLoS Medicine.

R2.3:

We thank reviewer 3 for his critical thoughts. However, we respectfully disagree with the overall judgment about our work. In fact, almost all studies evaluating risk of breakthrough infections or re-infections are based on population-data. Very few studies of our size have longitudinally recorded infection/vaccination history along with serology results to assess the baseline immune status. Another important difference to previous studies is that we considered behavior and exposure variables in our multivariable analysis. Although including these variables did not significantly change our results adjusting for booster vaccination only, we think that this aspect should be acknowledged. We have now highlighted these particular strengths of our study in the introduction and are again taking this up in the discussion section.
