# Supplementary material for: Risk and symptoms of COVID-19 in health professionals according to baseline immune status and booster vaccination during the Delta and Omicron waves in Switzerland—A multicentre cohort study
Source: PLoS Med. 2022 Nov 7;19(11):e1004125. doi: 10.1371/journal.pmed.1004125 (PMC9678290; doi:10.1371/journal.pmed.1004125)
Supplement: Attachment — Submitted filename: PMEDICINE-D-22-01834R3_RevisionNotes.pdf [file pmed.1004125.s015.pdf]

Kantonsspital St.Gallen

Kantonsspital St.Gallen

CH-9007 St.Gallen
Tel. 071 494 11 11
www.kssg.ch

Departement Innere Medizin
Infektiologie / Spitalhygiene
Prof. Dr. S. Kuster, Chefarzt

PLoS Medicine
D-22-01834R3

St.Gallen, October 5 2022

Point to Point Reply for Manuscript PMEDICINE-D-22-01834R2 entitled

“Risk and symptoms of COVID-19 in health professionals according to baseline immune status and booster vaccination during the Delta and Omicron waves in Switzerland: a multicentre cohort study”

Dear editors
Dear reviewers

We thank you again for the provisional acceptance of our manuscript. We have considered all comments and you will find below our point-to-point reply to the points raised.

Christian Kahlert and Philipp Kohler, in the name of all co-authors

Request from the editors

[1] Update manuscript title
Done

[2] Place de-identified data in public repository
Done. The data will become available freely to the public along with the publication of the manuscript. DOI https://dx.doi.org/10.5281/zenodo.7149075

[3] Revision of DAS.
Done

[4] Adapt methods based on response to reviewer #2, comment 3 (R2.3)
Done

[5] Confirmation involvement of Epitrack and comment on competing interest.
In order to clarify the commercial affiliations of some of the authors, we would like to declare that OLN worked on the project as a key professional in developing and implementing the digital platform. Beyond that, Epitrack provided support in the form of salaries for author OLN. This does not alter our adherence to PLOS Medicine publishing policies and study integrity.

[6,7] shorten the bullet point at lines 90-94 (Author Summary) and include number of participants
Done

[8] Line 150: '…we aimed to determine...'
Done

[9] include details of how informed consent was sough
Done

[10] confirm that the usage rights for this figure are compatible with PLOS Medicine's Creative Commons Attribution (CC BY) license
We have an active BioRender subscription (Academic, Individual) with publishing rights. CCBY Licence to publish this figure within the context of this manuscript is attached (below).

[11] ensure correct hyperlinking including supporting Information are labelled as described
Done

[12] define the groups in the legend of Figure S2
Figure S2 has been updated accordingly.

[13] move the reference to Table S2 from the Table 2 title to the legend, likewise for the corresponding reference in Table S2
Done

[14] observation design, please refer to associations rather than effects
Done, even beyond the lines mentioned.

[15] relocate the discussion of strenghts
Done

[16] By using a single imputed dataset, the analysis fails to take account of the added uncertainty in the results caused by the fact that some data are missing. By using multiple imputation, this uncertainty can be represented through the confidence intervals around the model parameters.
We have now re-ran all adjusted models using the confidence intervals derived from ten imputed datasets. Numbers have minimally changed. All numbers in the text and tables 2, S2-S8 have been updated accordingly. The following text has been added to the methods in the supplementary file:

"Ten series of imputations were performed, generating ten sets of covariate values, each of which was then combined with the outcomes and time-dependent covariates. Multivariable models were fitted separately to the ten data sets to obtain ten sets of model coefficients and standard errors. These were combined using the pooling rules implemented in the function "pool" of R package "mice". All results presented in tables of multivariable models are derived from the pooled statistics."

In addition to the above mentioned changes we have updated reference 14 (Overview on global SARS-CoV-2 variants).

BioRender Publication Licensing

The author **(Christian Kahlert)** has a Premium subscription to BioRender (biorender.com), and hereby BioRender grants **(PLOS Medicine)** the permission to publish the figure, and only figures:

S1 Figure. Definition of four groups by immune status and outcomes

On the manuscript **(Risk and symptoms of COVID-19 in health professionals according to baseline immune status and booster vaccination during the Delta and Omicron waves in Switzerland – a multicentre cohort study)** on CC BY 4.0 license. This includes the permission to publish the figure titled above in all formats including print and digital versions. The manuscript must include an acknowledgement of “Created with BioRender.com” on all versions of the publication to acknowledge that the figure has been created using BioRender.

This permission is exclusively granted for the figure titled above and does not supersede or modify in any way BioRender’s ownership, rights or license related to any of its intellectual property, as outlined in the BioRender terms and conditions located at https://biorender.com/terms/.

REP NAME, Customer Experience Representative

BioRender (Science Suite Inc.)

Mayet Awoke	October 6, 2022
Signature	Date
